# Age and sex corrected normal reference values of T1, T2 T2* and ECV in healthy subjects at 3T CMR

**DOI:** 10.1186/s12968-017-0371-5

**Published:** 2017-09-21

**Authors:** Clotilde Roy, Alisson Slimani, Christophe de Meester, Mihaela Amzulescu, Agnès Pasquet, David Vancraeynest, Jean-Louis Vanoverschelde, Anne-Catherine Pouleur, Bernhard L. Gerber

**Affiliations:** 1 Pole of Cardiovascular Research (CARD), Institut de Recherche Expérimentale et Clinique, Cliniques Universitaires St. Luc, Université Cathologique, Brussels, Belgium; 2 Division of Cardiology, Department of Cardiovascular Diseases, Cliniques Universitaires St. Luc UCL, Av Hippocrate 10/2806, B-1200 Woluwe St., Lambert, Belgium

**Keywords:** T1 mapping, T2 mapping, T2* mapping, Extracellular volume, 3 T, Normal values

## Abstract

**Background:**

Myocardial T1, T2 and T2* imaging techniques become increasingly used in clinical practice. While normal values for T1, T2 and T2* times are well established for 1.5 Tesla (T) cardiovascular magnetic resonance (CMR), data for 3T remain scarce. Therefore we sought to determine normal reference values relative to gender and age and day to day reproducibility for native T1, T2, T2* mapping and extracellular volume (ECV) at 3T in healthy subjects.

**Methods:**

After careful exclusion of cardiovascular abnormality, 75 healthy subjects aged 20 to 90 years old (mean 56 ± 19 years, 47% women) underwent left-ventricular T1 (3-(3)-3-(3)-5 MOLLI)), T2 (8 echo- spin echo-imaging) and T2 * (8 echo gradient echo imaging) mapping at 3T CMR (Philips Ingenia 3T and computation of extracellular volume after administration of 0.2 mmol/kg Gadovist). Inter- and intra-observer reproducibility was estimated by intraclass correlation coefficient (ICC). Day to day reproducibility was assessed in 10 other volunteers.

**Results:**

Mean myocardial T1 at 3T was 1122 ± 57 ms, T2 52 ± 6 ms, T2* 24 ± 5 ms and ECV 26.6 ± 3.2%. T1 (1139 ± 37 vs 1109 ± 73 ms, *p* < 0.05) and ECV (28 ± 3 vs 25 ± 2%, *p* < 0.001), but not T2 (53 ± 8 vs 51 ± 4, p = NS) were significantly greater in age matched women than in men. T1 (*r* = 0.40, *p* < 0.001) and ECV (*r* = 0.37, *p* = 0.001) increased, while T2 decreased significantly (*r* = −0.25, *p* < 0.05) with increasing age. T2* was not influenced by either gender or age. Intra and inter-observer reproducibility was high (ICC ranging between 0.81-0.99), and day to day coefficient of variation was low (6.2% for T1, 7% for T2, 11% for T2* and 11.5% for ECV).

**Conclusions:**

We provide normal myocardial T2, T2*,T1 and ECV reference values for 3T CMR which are significantly different from those reported at 1.5 Tesla CMR. Myocardial T1 and ECV values are gender and age dependent. Measurement had high inter and intra-observer reproducibility and good day-to-day reproducibility.

## Background

Cardiovascular magnetic resonance (CMR) myocardial tissue mapping techniques become more and more widely used in clinical practice. Indeed, they allow to pixel wise measurement of T1, T2 and T2* time constants in the heart and offer new opportunities for non-invasive identification of various cardiac infiltrative pathologies [1]. In particular, native T1 mapping has been proposed for detection of myocardial fibrosis, edema, amyloid, iron overload, and lipid accumulation (such as in Fabry disease) [2]. Comparison of pre and post T1 mapping allows estimation of extracellular volume (ECV), for quantification of interstitial fibrosis and identification of myocardial amyloid disease [3]. T2 mapping techniques allow detection of myocardial edema in acute myocardial infarction and myocarditis [4], estimation of area at risk [5] and intramyocardial hemorrhage [6] in acute infarction and have also shown potential for estimation of myocardial fibrosis in various cardiac diseases [7]. Finally, T2* mapping is established to diagnose and monitor the iron overload in cardiac and liver tissues [8]. It has also been used for diagnosis of intramyocardial hemorrhage [9]. Yet, use of these mapping techniques requires accurate knowledge of normal ranges and their influence on physiological parameters such as age and gender.

Whereas normal values for T1, T2 and T2* times are well established for 1.5 Tesla (T) CMR [8, 10–15], data for 3T remain scarce. Also the age and gender dependence of these normal values remains incompletely understood. Therefore we sought to determine normal reference values for T1, T2, T2* mapping and extracellular volume (ECV) at 3T in healthy subjects of different age groups and identify factors that are independently associated with T1, T2 and T2* relaxation time in these subjects. Furthermore, we also evaluated inter and intra-observer and day-to-day reproducibility aiming to determine how well these parameters are suited to serially follow patients over time.

## Methods

### Study population

The protocol was approved (#2014/19NOV/577 ‘VAL REF’) by the IRB of the Cliniques St. Luc UCL Brussels, Belgium and subjects were included after giving written informed consent to participate in this study. Healthy asymptomatic volunteers of different age groups (about 10 per decade) aged between 20 and 90 years were screened by advertisement in the local community. Prior to inclusion and CMR, all subjects underwent a clinical exam and assessment of medical history and cardio-vascular risk factors ie smoking (active smoking or history of >5UAP), hypertension (systolic and diastolic blood pressure > 140/90 mmHg during home controls or treatment), hypercholesterolemia (cholesterol >190 mg/dl or/and LDL cholesterol >115 mg/dl), history of familiar cardio-vascular disease (acute coronary syndrome or coronary revascularization in first degree relatives < 65 years old) and obesity (BMI > 30 kg/m2). All subjects underwent rest and stress ECG, 2D echocardiography and blood sampling with measurement of GFR (MDRD formula), hematocrit, cholesterol and NT-proBNP prior to inclusion.

Exclusion criteria were 1) any evidence of heart disease as indicated by clinical history, physical exam, atrial fibrillation, presence of abnormal rest or stress ECG or presence of abnormal cardiac function or valve disease at the echocardiography; 2) pregnancy; 3) contra-indications to CMR (ferrometallic cerebral aneurysm clips, pacemaker or implantable defibrillator, or severe claustrophobia), or to injection of gadolinium (Gd) based contrast agent (ie allergy to contrast media or renal insufficiency with GFR-MDRD < 30 ml/min/1.73 m2) and 4) diabetes.

Scan-rescan reproducibility of pre-, post- T1, T2 mapping, T2* and ECV was performed in 10 additional healthy subjects (60% female, mean age 33 ± 10 years) who underwent identical CMR exams on two consecutive days.

### CMR acquisition

CMR was performed using a 3 Tesla system (Ingenia, Philips Healthcare, Best, the Netherlands). To assess left ventricular (LV) myocardial function and mass, 12 consecutive 10 mm short-axis images and 2-, 3- and 4-chamber long axis image of the LV were acquired using a cine balanced steady state free precession sequence (bSSFP). Then, one mid-ventricular short axis and one 4 chamber Modified look locker (MOLLI) images were acquired for T1 determination using an 11 image, 17 heart-beat 3-(3)-3-(3)-5 bSSFP sequence [16]. Imaging parameters were: field of view (FOV): 340 mm, slice thickness 8 mm, slice gap 0 mm, flip angle 35 degrees, repetition time (TR): 2.6 ms echo time (TE) 1.03 ms, matrix 172 × 150 pixels resulting in a resolution of 2 × 2.6 mm, SENSE factor 2, trigger delay end-diastole, inversion times ranging from 150 to 3287 ms. T2 mapping was performed using a multiecho Gradient-spin-echo (GRASE) sequence on the same midpapillary ventricular short-axis slice as T1 mapping. TR was one RR interval. Eight echos were acquired using echo time 8-64 ms and Echo-train length 40. Slice thickness was 10 mm, matrix 196 × 140 pixels and FOV 320 × 320 mm. Subsequently in the same location, myocardial T2* was assessed from a midpapillary ventricular short-axis slice with a single breath hold segmented, multi-echo gradient echo sequence using 8 echoes with a minimum TE of 2.0 ms, an echo spacing of 2.2 ms and a TR of 19.1 ms.

Then, a total dose of 0.2 mmol/kg gadobutrol (Gadovist, Schering) was injected in a 2 phase protocol: 3 cm^3^ gadobutrol were infused as bolus pushed by a 15 cm^3^ saline at 3 cm^3^/s, 15 s later, the remaining contrast dose followed by 20 cm^3^ saline were infused at a slower rate of 1 cm^3^/s. Ten minutes after contrast injection, short- and long-axis 2D inversion recovery late gadolinium enhancement (LGE) images were acquired with an inversion-recovery gradient-echo imaging sequence to evaluate focal myocardial fibrosis. Finally, post-contrast MOLLI T1 mapping was repeated in identical prescription as pre-contrast T1 mapping, 12 min after gadolinium injection. This time was consistent across subjects.

### CMR analysis

CMR images were anonymized and analyzed in double by two experienced observers (CR and AS with 3 years of CMR experience and level 2 Euro-CMR certification) blinded to clinical data.

Cine bSSFP CMR images were analyzed using the freely automated software Segment v1.9 (http://segment.heiberg.se) [17]. The endocardium and epicardium of the left ventricle were fully automatically contoured on all phases of the left ventricle, with manual adjustments when needed. Left ventricular end-diastolic volume (LV-EDV) and end-systolic volume (LV-ESV) were calculated using summation of discs method. The first image of the cardiac cycle was considered to be end-diastole whereas the smallest volume of the LV curve was considered end-systolic volume. LV mass was computed assuming a myocardial density of 1.06 and excluding papillary muscles. LV volumes and mass were indexed to body surface area. LVEF was computed as (EDV-ESV)/EDV.

**Fig. 1:**
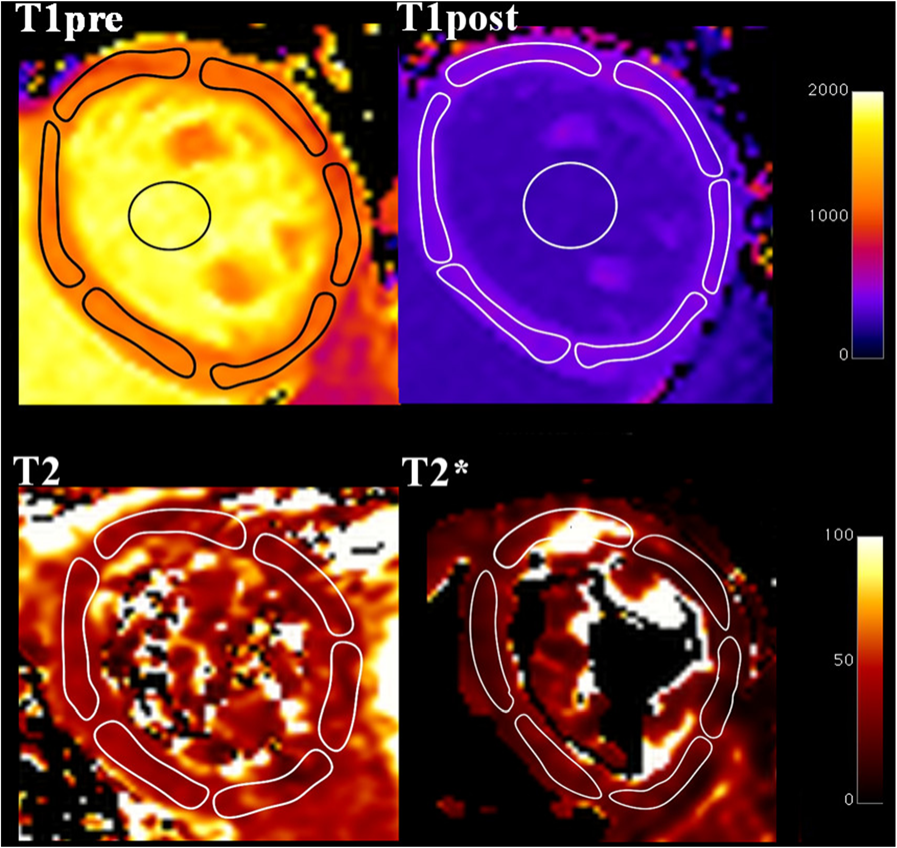
Example of native and post contrast T1 mapping, T2 and T2* maps in a healthy volunteer

Pixelwise T1 maps were generated using the open-source software MRmap v1.4 [18] under IDL. Images were corrected for respiratory motion when needed. T1 maps were generated by fitting pixels to the equation s(t) = a – b exp. (t/T1*), and T1 = T1*((b/a-1), where a and b are constants, t is time and s(t) signal intensity at time t. T2 maps were created by a T2 plugin in Osirix, while T2* maps were generated on the scanner by respectively monoexponential fit to the equations y = K e ^-TE/T2^ and y = K e ^-TE/T2*^. The T1, T2 and T2*maps were saved in DICOM format and imported into Osirix software (Pixmeo; Switzerland; version 5.8). Pre and post contrast blood T1 times were measured on a region of interest manually drawn in the center of the blood pool. Pre and post contrast T1, T2, T2* time values were measured in a 6 ROI’s per slice in the short axis view (Fig. 1) and were reported as value for the septum and the entire heart. In 32 subjects, T1 time and ECV were also measured in a septal ROI in 4 chamber view. The partition coefficient lambda (λ) and ECV were computed as:

$$ \boldsymbol{\lambda} =\frac{1/ T1 myocardium\  postC-1/ T1 myocardium\  preC}{1/ T1 blood\  postC-1/ T1 blood\  preC} $$

$$ \boldsymbol{ECV}=\left(1- Hematocrit\right)\bullet \lambda $$

Hematocrit measurements were performed either on the day, or one day after the CMR study.

### Statistical analysis

Statistical analyses were performed using SPSS ( version 21.0 software International Business Machines, IBM Inc., Chicago, IL). Continuous variables were expressed as mean ± one standard deviation (SD) or medians [quartiles] if not normally distributed; categorical variables were reported as counts and percentages. All tests were 2 sided and *p* value <0.05 was considered statistically significant.

Subjects were categorized into different decades. Categorical variables of subjects in different age groups were compared using χ^2^ or exact test. Continuous variables were compared among groups using ANOVA when normally distributed, else using Kruskall-Wallis test. Individual differences among groups were compared post-hoc using Mann-Whitney U tests (with Bonferroni correction for multiple comparisons) for non-normally distributed data and Tukey-Kramers test for normally distributed data with equal variances. Differences between 4 chamber and short-axis T1 and ECV were measured using paired T test. Relations of T1, T2 and T2* times and ECV with age were compared using Pearson’s correlation coefficient. Male and female subjects were matched for age thanks to a propensity score (1:1) with a caliper of 0.25 standard deviations of the propensity score, using the STATA 10.0 software (StataCorp LP, College Station, Tex) and the psmatch routine. Uni and multiple regression analysis was used to assess the effects of clinical characteristics on T1 times, ECV, T2 mapping and T2* times. Intra and inter observer and day-to-day reproducibility of T1 T2 T2* times and ECV were assessed with an intraclass correlation coefficient and using coefficient of variation (SD/Mean).

## Results

### Study population

Seventy six healthy subjects were screened, 1 was excluded due to presence of diabetes. The final population consisted of 75 participants. The baseline characteristics of the 75 subjects are shown in Table 1. All participants were Caucasian. Systolic blood pressure increased while GFR decreased with age. Fifty five volunteers (73%) had at least one cardiovascular risk factor. Prevalence of CV risk factors, in particular hypertension and hypercholesterolemia, increased with age. No subject had history of stroke, chronic obstructive pulmonary disease or sleep apnea syndrome. Also no subject presented hemochromatosis or anemia.

**Table 1:** Patients characteristics

	Total population *n* = 75	[20–30] *n* = 10	[30-40] *n* = 9	[40-50] *n* = 9	[50-60] *n* = 11	[60-70] *n* = 11	[70-80] *n* = 20	>or = 80 *n* = 5	*P* value
Age (years)	56 ± 19	26 ± 3	36 ± 3	45 ± 3	55 ± 3	64 ± 3	74 ± 3	84 ± 5	<0.001
Weight (kg)	71 ± 13	66 ± 11	75 ± 13	71 ± 10	71 ± 12	75 ± 16	69 ± 11	74 ± 18	0.63
Height (m)	1.71 ± 0.08	1.76 ± 0.06	1.76 ± 0.06	1.72 ± 0.07	1. 68 ± 0.08	1.75 ± 0.07	1.65 ± 0.07	1.68 ± 0.10	0.04
BMI (kg/m^2^)	24 ± 4	21 ± 2	24 ± 4	24 ± 3	25 ± 4	24 ± 4	25 ± 3	26 ± 5	0.12
BSA (m^2^)	1,8 ± 0,2	1.8 ± 0.2	1,9 ± 0,2	1,8 ± 0,1	1,8 ± 0,2	1,9 ± 0,2	1,8 ± 0,2	1,8 ± 0,3	0.31
Sex (Female)	36 (48%)	4 (40%)	2 (22%)	4 (44%)	5 (45%)	4 (36%)	14 (70%)	3 (60%)	0.27
SBP (mmHg)	133 ± 20	120 ± 17 ε	122 ± 20ε	126 ± 12	136 ± 12	133 ± 14	145 ± 23†¥	144 ± 17	0.003
HR (bpm)	67 ± 10	67 ± 9	68 ± 7	67 ± 11	67 ± 15	69 ± 9	68 ± 9	63 ± 8	0.95
GFR (ml/min/1.73m^2^)	77 ± 16	88 ± 19 ε◊	88 ± 11$ε◊	80 ± 12	76 ± 8	70 ± 9¥	72 ± 19†¥	66 ± 12†¥	0.006
Hematocrit (%)	42 ± 3	43 ± 3	44 ± 2	41 ± 2	42 ± 3	42 ± 2	42 ± 5	39 ± 2	0.71
Ferritin (μg/l)	120 [15;619]	167 [35;294]	118 [26;591]	129 [28;257]	129 [21;196]	115 [15;182]	126 [16;619]	104 [54;126]	0.64
Smoking (%)	9 (12%)	0 (0%)	0 (0%)	1 (11%)	0 (0%)	2 (18%)	5 (25%)	1 (20%)	0.26
Hypertension (%)	19 (25%)	0 (0%)	0 (0%)	0 (0%)	1 (9%)	2 (18%)	10 (50%)	5 (100%)	0.001
Hypercholesterolemia (%)	46 (60%)	2 (20%)	2 (22%)	4 (44%)	8 (73%)	7 (64%)	18 (90%)	4 (80%)	0.001
Family history of CAD (%)	10 (13%)	1 (10%)	3 (33%)	0 (0%)	2 (18%	1 (9%)	1 (5%)	1 (20%)	0.19

† .vs 20-29y; ¥ .vs 30-39y; ǂ .vs 40-49y; * .vs 50-59y; $. Vs 60-69y; ε. vs 70-79y; ◊.vs ≥ 80

### CMR protocol

The CMR protocol was successfully completed in all subjects. All participants had normal cardiac function and no focal scar on LGE images.

Artifacts precluded analysis of native T1 in 1 patient and of post contrast T1 maps in 2 subjects, resulting in missing ECV in 3 subjects. T2 and T2* maps were not analyzable due to artifacts in 1 subject.

### Normal CMR values and relation to age and sex

**Fig. 2:**
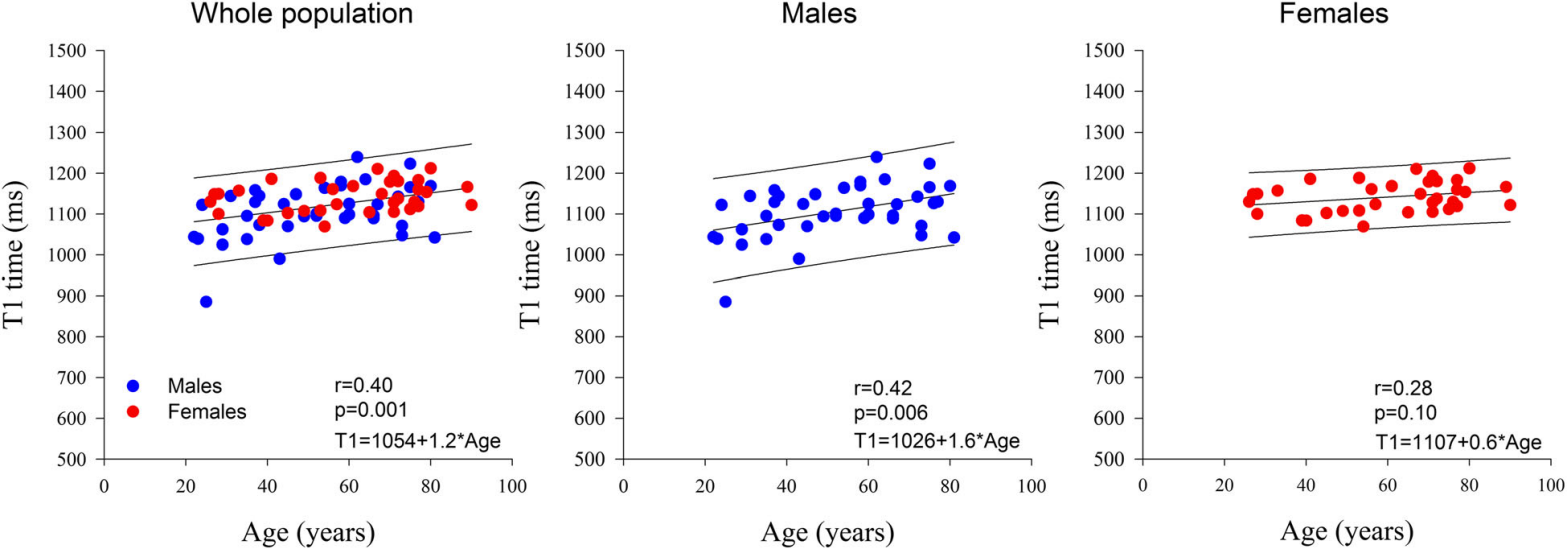
Native T1 time according to age and sex

**Fig. 3:**
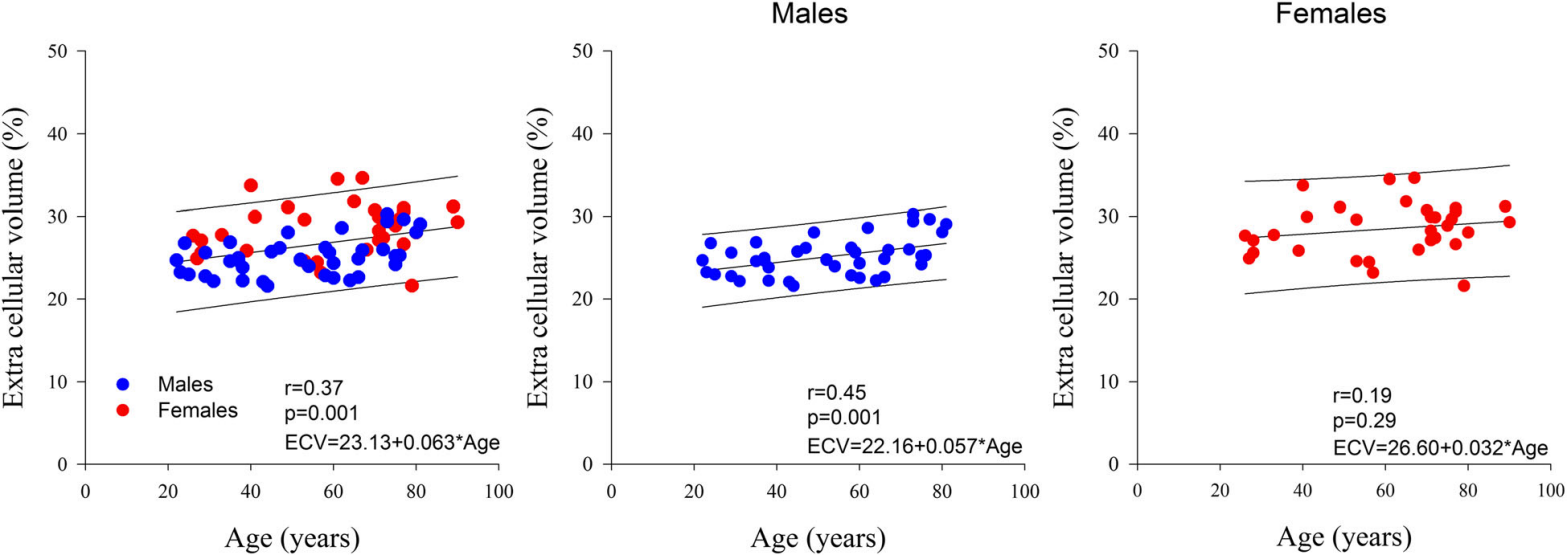
ECV according to age and sex

**Fig. 4:**
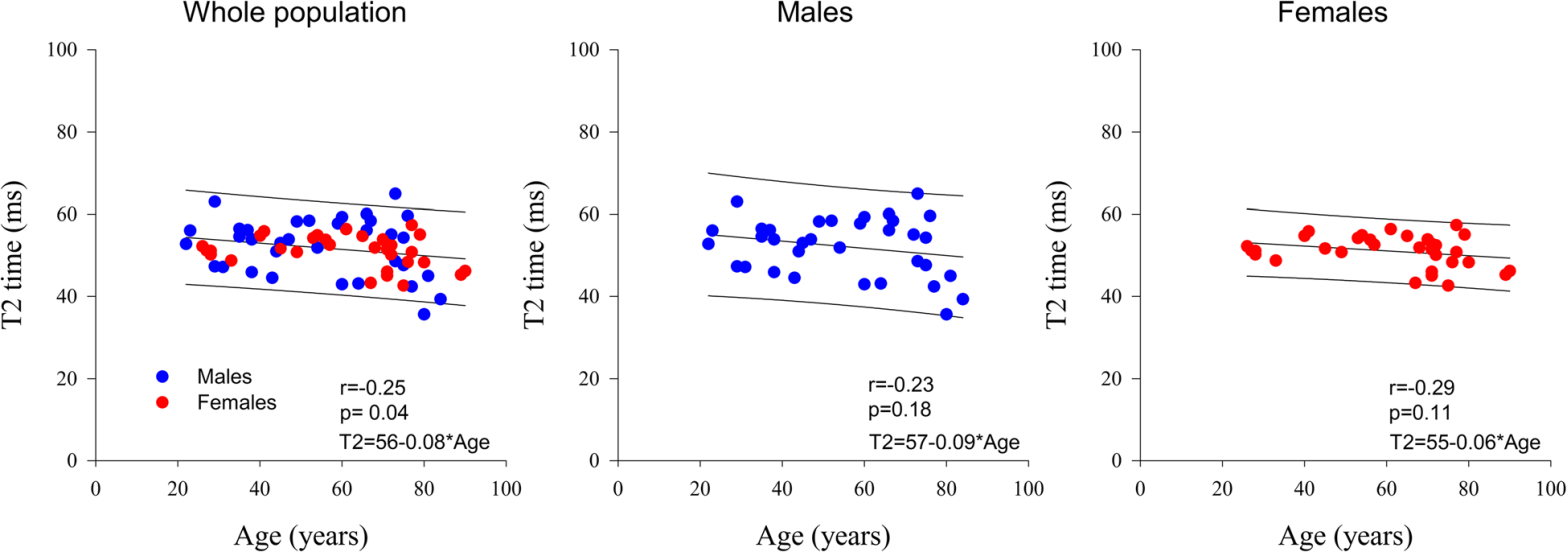
T2 time according to age and sex

**Fig. 5:**
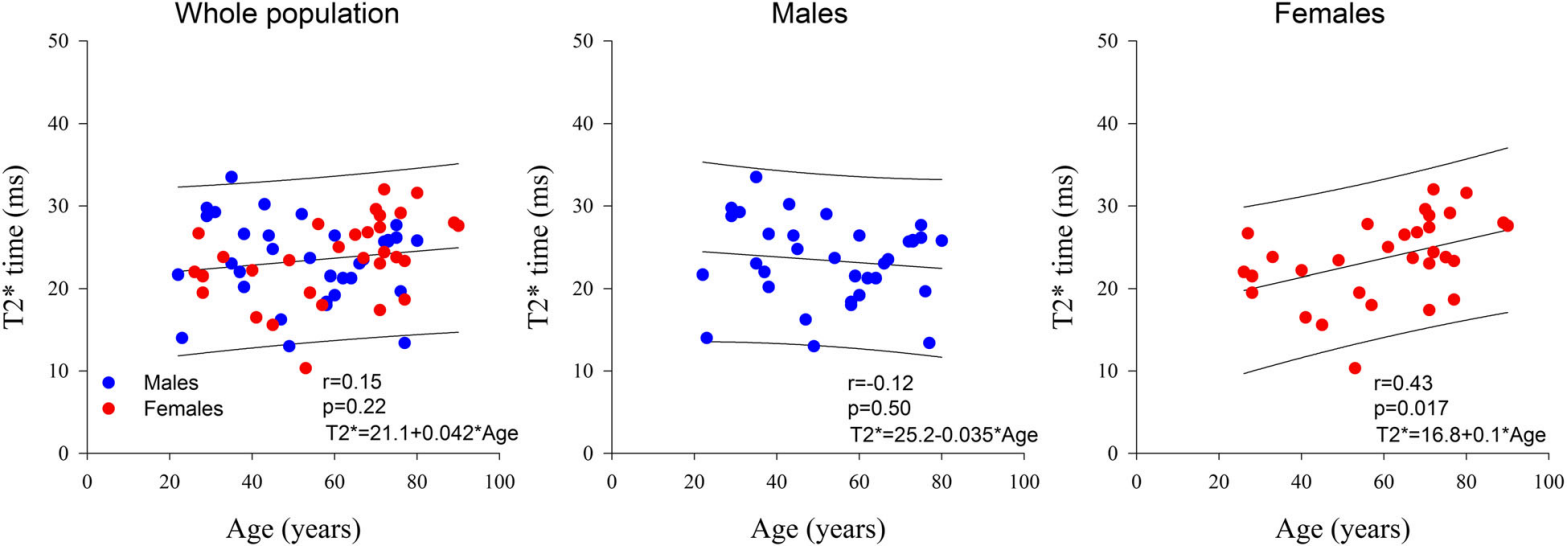
T2* time according to age and sex

Normal CMR reference values of the 75 subjects according to decade age groups are shown in Table 2. Both indexed LV and RV end-diastolic and end-systolic volumes decreased, while LV and RV ejection fraction increased significantly with increasing age. LV mass to volume ratio also increased significantly with age. The individual values of native T1, ECV, T2 and T2* values and their relation to age are shown in Table 2 and Figs. 2, 3, 4 and 5. Native T1 time and whole myocardium ECV significantly increased, whereas T2 decreased significantly with age. By contrast, T2* was not influenced by age. Age related correlation of T1 and ECV was present however only in male and not in female subjects, while age related correlation of T2 were not any more significant individually in either gender subgroup.

**Table 2:** CMR parameters according to age

		Total population *n* = 75	[20–30] *n* = 10	[30-40] *n* = 9	[40-50] *n* = 9	[50-60] *n* = 11	[60-70] *n* = 11	[70-80] *n* = 20	>or = 80 *n* = 5	*P* value
LV EDVi (ml/m2)		79 ± 17 [48;119]	98 ± 11ǂ*ε◊ [87;118]	93 ± 14ε◊ [72;121]	82 ± 12†ε◊ [60;102]	78 ± 13†ε◊ [65;107]	82 ± 15ε◊ [57;102]	66 ± 12†¥ǂ*$ [46;91]	59 ± 7†¥ǂ*$ [50;66]	<0.001
LV ESVi (ml/m2)		29 ± 9 [14;50]	40 ± 7*$ε◊ [32;50]	34 ± 8ε◊ [18;45]	32 ± 8ε◊ [21;45]	28 ± 7†◊ [18;42]	29 ± 8†◊ [16;42]	24 ± 6†¥ǂ [15;35]	17 ± 5†¥ǂ*$ [11;25]	<0.001
LV-EF (%)		64 ± 6 [55;76]	59 ± 3◊ [54;63]	64 ± 5 [57;75]	61 ± 5◊ [55;72]	64 ± 6 [55;75]	66 ± 6 [57;76]	65 ± 4 [55;68]	72 ± 6†ǂ [62;78]	0.03
LV mass i (g/m2)		58 ± 11 [36;81]	56 ± 9 [44;72]	58 ± 9 [49;78]	54 ± 15 [35;68]	56 ± 7 [46;67]	64 ± 12 [44;82]	59 ± 13 [36;79]	60 ± 11 [48;70]	0.56
Mass/Vol ratio (%)		0.8 ± 0.2 [0.5;1.2]	0.6 ± 0.05$ε◊ [0.5;0.7]	0.6 ± 0.1ε◊ [0.5;0.8]	0.7 ± 0.2 ε◊ [0.4;0.9]	0.7 ± 0.1 ε◊ [0.6;0.8]	0.8 ± 0.1†◊ [0.7;1.0]	0.9 ± 0.1†¥ǂ* [0.7;1.2]	1.0 ± 0.3†¥ǂ*$ [0.8;1.4]	<0.001
RV-EDVi (ml/m2)		82 ± 18 [52;132]	104 ± 17ǂ*ε◊ [83;132]	95 ± 17ε◊ [71;133]	83 ± 12† [63;105]	78 ± 12† [64;105]	87 ± 18ε◊ [67;129]	69 ± 12†¥$ [51;92]	68 ± 11†¥$ [57;80]	<0.001
RV-ESVi (ml/m2)		35 ± 10 [19;63]	47 ± 10ǂ*$ε◊ [35;64]	40 ± 8ε◊ [26;55]	37 ± 4†ε◊ [30;45]	32 ± 8† [24;44]	38 ± 8†ε◊ [25;52]	28 ± 7†¥ǂ$ [16;40]	25 ± 6†¥ǂ$ [19;34]	<0.001
RV EF (%)		58 ± 5 [48;68]	55 ± 3*ε◊ [49;59]	58 ± 4 [50;63]	55 ± 2*ε◊ [52;58]	59 ± 5†ǂ [49;67]	57 ± 3◊ [52;63]	59 ± 6†ǂ [47;75]	63 ± 5†ǂ$ [56;66]	0.007
T1 (ms)	Whole myoc	1122 ± 57 [977;1225]	1070 ± 80$ε◊ [885;1149]	1114 ± 43 [1038;1158]	1100 ± 54 [990;1186]	1132 ± 42 [1069;1188]	1144 ± 51† [1089;1239]	1141 ± 42 [1047;1223]	1142 ± 64† [1042;1212]	0.01
	septum	1162 ± 81 [954;1285]	1066 ± 133 [768;1209]	1141 ± 76 [987;1233]	1142 ± 78 [991;1225]	1182 ± 40 [1099;1232]	1182 ± 60 [1108;1305]	1196 ± 42 [1094;1266]	1188 ± 75 [1081;1282]	0.01
ECV %	Whole myoc	26.6 ± 3.2 [21.6;34.5]	25.1 ± 1.7 [22.7;27.7]	24.8 ± 1.9 [22.2;27.7]	27.3 ± 4.3 [21.6;33.7]	25.0 ± 1.9 [22.9;29.6]	27.1 ± 4.6 [22.2;34.6]	28.0 ± 2.6 [21.6;31.0]	29.1 ± 1.3 [28.1;31.2]	0.01
	septum	27.5 ± 3.8 [21.0;37.6]	26.7 ± 1.3 [24.5;28.3]	26.4 ± 4.2 [21.0;35.0]	28.9 ± 6.0 [21.7;40.3]	25.9 ± 1.9 [22.6;29.7]	26.4 ± 4.2 [21.0;34.4]	28.7 ± 3.8 [21.9;36.9]	29.9 ± 1.8 [27.4;32.5]	0.19
T2 (ms)	Whole myoc	52 ± 6 [41;61]	53 ± 5 [48;62]	52 ± 4 [46;56]	53 ± 4 [46;58]	55 ± 2 [52;58]	53 ± 7 [43;60]	52 ± 6 [42;63]	43 ± 5 †¥ǂ*$ε [36;48]	0.007
	septum	52 ± 7 [38;64]	54 ± 4 [49;59]	53 ± 4 [48;59]	54 ± 4 [47;61]	56 ± 4 [52;63]	53 ± 9 [38;64]	51 ± 6 [41;62]	42 ± 5†¥ǂ*$ε [34;46]	0.004
T2* (ms)	Whole myoc	24 ± 5 [12;33]	23 ± 5 [14;30]	26 ± 5 [20;34]	21 ± 6 [13;30]	21 ± 6 [10;29]	24 ± 3 [19;27]	25 ± 5 [13;32]	28 ± 3 [26;32]	0.27
	septum	25 ± 5 [11;39]	27 ± 6 [22;39]	26 ± 4 [22;31]	23 ± 8 [11;39]	22 ± 7 [11;31]	24 ± 5 [16;35]	25 ± 4 [19;35]	28 ± 2 [24;31]	0.38

Data are reported as mean ± SD and 95% IQR

† *p* < 0.05 .vs 20-29y; ¥ .vs 30-39y; ǂ .vs 40-49y; * .vs 50-59y; $. Vs 60-69y; ε. vs 70-79y; ◊.vs ≥ 80 & *p* < 0.05 septum vs whole myocardium of the same group

Using propensity score matching, 28 male and female subjects were matched for age. Sex related differences of CMR parameters are shown in Table 3. Female subjects had lower BMI and BSA (22 ± 3 kg/m^2^ vs. 25 ± 3 kg/m^2^, *p* = 0.002 and 1.66 ± 0.11 m^2^ vs. 1.93 ± 0.13 m^2^, *p* < 0.001 respectively), lower hematocrit (41 ± 2% vs. 44 ± 4%, *p* < 0.001) but no significant difference in cardiovascular risk factors or ferritin level. By CMR, females had lower indexed LV mass, lower indexed LV and RV end-diastolic volume and lower mass/volume ratio than males. LV and RV ejection fraction were similar. Native T1 septal times (with a trend for whole myocardial T1 time) and ECV were significantly higher in women than in men. Whole T2 times and T2* were not different. Native blood (*r* = −0.24, *p* < 0.02), but not myocardial T1 times (*r* = 0.001, p = NS) were weakly correlated to hematocrit.

**Table 3:** CMR parameters according to gender in 56 propensity score matched patients of similar age

		Female *n* = 28	Male *n* = 28	*P* value
Age (years)		55 ± 17	56 ± 18	0.82
BMI (Kg/m^2^)		22 ± 3	25 ± 3	0.002
BSA (m^2^)		1.66 ± 0.11	1.93 ± 0.13	<0.001
SBP (mmHg		129 ± 20	138 ± 20	0.12
HR (bpm)		62 ± 7	64 ± 11	0.51
GFR (ml/min/1.73m^2^)		73 ± 16	77 ± 13	0.28
Hematocrit (%)		41 ± 2	44 ± 4	0.008
Smoking (%)		3 (11%)	3 (11%)	1.00
Hypertension (%)		6 (21%)	7 (25%)	0.77
CV familial history (%)		2 (7%)	5 (18%)	0.23
Hypercholesterolemia (%)		17 (61%)	16 (57%)	0.79
LV EDVI(ml/m2)		75 ± 14	84 ± 17	0.039
LV ESVI(ml/m2)		27 ± 7	31 ± 10	0.07
LV EF(%)		64 ± 4	63 ± 6	0.48
LVMI(g/m2)		51 ± 10	64 ± 9	<0.001
LV_Mass/Volume ratio		0.7 ± 0.1	0.8 ± 0.2	0.017
RVEDVI(ml/m2)		77 ± 15	88 ± 18	0.011
RVESVI (ml/m2)		31 ± 8	39 ± 9	0.005
RVEF(%)		59 ± 5	57 ± 5	0.09
T1 Pre contrast (ms)	Whole myoc.	1139 ± 38	1109 ± 73	0.054
	Septum	1194 ± 48	1128 ± 103	0.004
ECV %	Whole myoc.	28.4 ± 3.4	25.1 ± 2.2	<0.001
	Septum	28.8 ± 3.1	26.6 ± 4.4	0.044
T2 time (ms)	Whole myoc.	51 ± 4	53 ± 8	0.28
	Septum	52 ± 5	54 ± 8	0.23
T2* time (ms)	Whole myoc.	23 ± 5	24 ± 5	0.55
	Septum	25 ± 5	24 ± 6	0.78

Multivariate regression models correcting T1, T2 and ECV values for Age and sex are shown in Table 4.

**Table 4:** Multivariate regression models for predictors of T1, ECV, and T2

Parameter	Coefficient	p
a) T1		
Intercept	975	<0.001
Age (years)	0.97	<0.005
Sex (female)	24	0.05
Heart Rate (bpm)	1.29	0.06
b) ECV		
Intercept	22.7	<0.001
Age (years)	0.042	0.014
Sex (female)	3.18	<0.001
c) T2		
Intercept	56	<0.001
Age (years)	−0.074	0.04

Age, Sex, systolic blood pressure, Heart Rate, GFR, and cardiovascular risk factors (smoking; hypertension, familary history of CAD and cholesterol level) were entered in the models

For 32 subjects, native T1 time and ECV were compared in a septal ROI in a short axis and a 4 chamber views. The mean 4C native T1 times and ECV were 1158 ± 80 ms and 27.4 ± 3.6% respectively. Both were non-significantly different from values obtained in the septum in the SA view (*p* = 0.40 and *p* = 0.73 respectively).

### Intra and inter-observer variability and day to day reproducibility of measurements

Intra and inter-observer reproducibilities for T1, ECV, T2 and T2* are shown in Table 5. All parameters had excellent intra- and inter-observer variability Day to day reproducibility of native T1, ECV, T2 mapping and T2* mapping in the 10 subjects undergoing test-retest was also high as evidenced by low coefficient of variation (Table 5).

**Table 5:** Day to day reproducibility and inter and intraobserver reproducibility of native T1, T2 T2* and ECV

	Interobserver reproducibility (ICC)	Intraobserver reproducibility (ICC)	Day 0	Day1	CV
Native T1 (ms)	0.96	0.97	1200 ± 84	1179 ± 64	6.2%
ECV (%)	0.87	0.74	29.8 ± 4.3	31.1 ± 2.5	11.5%
T2 (ms)	0.83	0.81	46 ± 3	47 ± 3	7%
T2 * (ms)	0.94	0.93	23.6 ± 3.0	24.1 ± 2.2	10.8%

## Discussion

Our study reports normal values of cardiac T1, T2, T2* and ECV values in healthy subjects aged 20-90 at 3T and examined their age and gender dependence.

T1, T2 and T2* time constants are fundamental magnetic characteristics which depend on tissue composition and field strength [19]. However measurements in vivo may also be influenced by the type of pulse sequence, scanner fitting algorithm and population. The reported normal ranges of our study allow for better detection of pathological states and contribute to the increased degree of standardization in CMR. While several earlier studies reported the normal range of T1, T2 and ECV in healthy subjects, most of these studies were performed at 1.5 T and no study reported all of these values in the same population. Recently, 3T scanners become more and more used for CMR, but reference values for 3T remain scarce. Table 6 compares our normal values of T1, T2 and T2* to those previously reported [10, 20–22]. As in vitro myocardial T1 measurements are approximately 30-40% higher at 3T than at 1.5 T, our values at 3T were higher than reference values at 1.5 T, but relatively similar to those reported by von Knobelsdorf at 3T [10, 23] whereas Dabir et al. [11] reported shorter and Kawel et al., using 8 image short MOLLI [20] longer T1 times than we.

**Table 6:** Comparison with reported T1, T2, T2* and ECV values at 1.5 and 3 T

	Present Study	Reported Values	
		1.5 T	3.0 T
T1 (ms)	1124 ± 57	950 ± 21 (*n* = 34) [12]†	1159 ± 41 (*n* = 60) [23]†
			1052 ± 23 (*n* = 32) [12]†
			1286 ± 59 (*n* = 28) [20]
T2 (ms)	52 ± 6	GraSE:	GraSE:
		59 ± 4 (*n* = 30) [13]†	54 ± 4 (*n* = 30) [11, 13]
		T2-SSFP	T2-SSFP
		52 ± 3 (*n* = 14) [12]	44 ± 3 (*n* = 30) [11, 13]†
		53 ± 3 (*n* = 30) [11, 13]	45 ± 3 (*n* = 60) [23]†
T2* (ms)	25 ± 7	30 ± 7 (*n* = 10) [8]	20.5 (*n* = 20) [27]
		32 ± 2 (*n* = 30) [14]†	23 ± 2 (*n* = 20) [22]
		36 ± 5 (*n* = 30) [15]†	
ECV (%)	27 ± 3	25 ± 4 (*n* = 34) [12]†	26 ± 4 (*n* = 32) [12]

† *p* < 0.01 using paired t-test vs present study (performed only for group sizes *n* > 30)

As reported by Dabir et al. [10], but in contrast to Kawel et al. [24], our ECV values were also slightly higher than those reported for 1.5 T CMR, suggesting that interpretation of normal values of these values needs to take into account field strength. It could however be possible that these differences result from differences in contrast dose or timing.

Myocardial T2 times in vitro are approximately 20% higher at 3T than 1.5 T. However in vivo T2 measurements depend also on the type of pulse sequence, with GraSE giving longer measurements than T2 prep SSFP [13]. Indeed our T2 values at 3T were similar to those reported using GraSE by Baessler et al. [13], but higher than those reported using T2 prep SSFP by von Knobelsdorff et al. [23].

Further, our study is the largest to describe normal T2* values at 3T since there is paucity of data on T2* measurements at 3T [22, 25–27]. T2* values in our study at 3T were similar to those reported by Meloni et al. [22] but slightly higher than those reported by Alam et al. [21].

An interesting finding of our study was age and sex dependency of myocardial T1 times and ECV. We found that T1 and ECV increase with age in males, but not in females. Also T1 and ECV were overall higher in females than in males. A potential explanation could be age dependent increase of interstitial myocardial fibrosis in males but not females, as demonstrated by histopathology [28]. In the literature the relation between T1 and ECV and age is conflicting. Indeed age-dependent increase of T1 and ECV was also shown in two small series [24, 29] and in the large MESA study [30]. By contrast, Dabir et al. reported only a trend of a positive association between native T1 and age at 1.5 T in males [10], and Liu reported no significant influence of age on T1 [31]. In discrepancy, Piechnik et al. [32] showed no age relation between pre-contrast T1 and age in males and an inverse relation between T1 and age in females. Also the influence of gender on T1 and ECV remains debated. Indeed, both Piechnik et al. [32] and the MESA study [2] reported higher T1 and respectively both higher T1 and ECV values in females than in males. By opposition, Dabir [10] found no gender differences of T1 and ECV at 1.5 T in general population, and respectively Liu et al. [31] found no gender differences of native T1 at 3T in Afro-American population. In our study, gender differences in T1 and ECV persisted after adjustment for age and heart rate. The findings of our study, were thus very similar to those reported by Liu in the MESA trial.

Our study also found that T2 values were age dependent but not influenced by gender. These findings are in some disagreement with those reported by Bönner et al. [33] at 1.5 T, who demonstrated higher T2 values in females and age related increase rather than decrease of T2 value, and with those reported by von Knobelsdorff et al. [23], who did not find either age and sex dependency of T2 values in healthy volunteers.

Our study thus suggests that age and gender must be taken into account when interpreting T1,T2 and ECV mapping results in the heart. By contrast, T2* values do not need to be corrected for these parameters as they were neither influenced by age or gender.

The influence of several other parameters on T1 values, such as location of region of interest, cardiac phase [20], triglycerides, heart rate, and BMI had previously been reported [31]. However, this was not corroborated by our present work. Indeed we found only a minimal effect of heart rate on native T1 times, but not on ECV, T2 and T2* values, simplifying interpretation of results.

To evaluate the robustness of mapping techniques, our study reported not only inter-observer and intra-observer variability of T1, T2, T2* and ECV but also the day-to-day variability of all measurements. While test-retest reproducibility for native T1 [34], T2 [11] and T2* [21] have been reported, reproducibility was reported only for 1.5 T and not 3 T, and no prior study reported day-to-day variability of ECV so far. Reproducibility of all these measurements was high, confirming their usefulness to serially follow patients over time in particular for 3T CMR.

Finally, our series also report age and gender corrected values for LV and RV volumes and LV mass. In agreement with previously reported series [35, 36], we found that LV and RV volumes decrease with age, and are smaller in females than males. No age related effect on LV mass was found in our study, but indexed LV mass was lower in females than males.

### Limitations

This is a single center, single vendor study of moderate sample size. While we attempted to recruit 10 subjects of different gender per decade, we could not achieve exact gender matching for each age group, in particular for subjects >80 years old where it was difficult to find male subjects without cardiovascular history. Therefore, we used multivariate analysis and propensity matching to compare male and female subjects of similar age. Our population was 100% Caucasian, and values for other ethnicities were not evaluated.

The use of mapping sequences, particularly for T2* analysis at 3T may be hampered by more artifacts than at 1.5 T. Artefacted segments were therefore excluded from analysis. Also we performed measurements only in a single short axis slice. While we found no differences between measurements of T1 and ECV performed in 4 chamber and short axis orientation, we did evaluate T2 and T2* measurements in 4 chamber view, nor did we evaluate measurements in apical or basal locations. Myocardial tissue times may also differ for different vendors or different pulse sequences as well as different fitting algorithms, and normal values should probably be locally verified for each center. Due to longer T1 times at 3 T, T1 recuperation may not be fully complete for 3-(3)-3-(3)-5 Molli schemes, causing underestimation of T1 values at higher heart rates. MOLLI schemes ensuring full recovery between inversion pulses such as 5 s-(3 s)-3 s might provide more accurate T1 estimation at 3T and high heart rates, but were not yet available in our study. Since we observed a non-significant trend for increase rather than decrease of T1 at higher heart rate, we do not believe that this effect may have significantly affected our results. Also, we employed 35° flip angle which is the standard at 1.5 T, while others used 20° at 3 T, and this might introduce reproduciblilty issues. In our study, standard deviation of native T1, T2 and ECV values were somewhat larger than those previously reported. Since we found that these values are age dependent, this may be explained in part by wider age range of our population. Other possible explanations are reduced B0 and B1 field homogeneity at 3T then at 1.5 T. For T1, the larger variation may also be explained by the overall higher T1 values at 3 T. Also ECV values were determined after 0.2 mmol/kg gadubutrol, and slightly lower ECV values have been reported when lower doses of contrast media were injected.

Finally for day to day reproducibility of ECV, hematocrit values were sampled only once. Potential physiological day to day variation of hematocrit values were not thus not taken into account in the day-to-day variability of these measurements.

## Conclusions

We report specific normal myocardial T1, T2, T2*, and ECV reference values for 3T CMR to facilitate interpretation of CMR mapping techniques at this field strength. We demonstrate that measurements values at 3T were significantly different from those reported at 1.5 Tesla. Furthermore we found that T1 and ECV were age and sex dependent, while T2 was age dependent pnly, suggesting that it is necessary to adjust for age and sex when interpreting normal ranges of T1, T2 and ECV values for clinical CMR examination. Finally we demonstrated high inter- intra- and particularly day- to day- reproducibility of these measurements, indicating the ability of serially following these parameters over time.

## Abbreviations

BMI: Body mass index
BSA: Body surface area
ECV: Extracellular volume
EDVi: Indexed end diastolic volume
EF: Ejection fraction
ESVi: Indexed end systolic volume
LV: Left ventricle
RV: Right ventricle
